# Cinnamaldehyde-Based Self-Nanoemulsion (CA-SNEDDS) Accelerates Wound Healing and Exerts Antimicrobial, Antioxidant, and Anti-Inflammatory Effects in Rats’ Skin Burn Model

**DOI:** 10.3390/molecules27165225

**Published:** 2022-08-16

**Authors:** Kamal A. Qureshi, Salman A. A. Mohammed, Omar Khan, Hussein M. Ali, Mahmoud Z. El-Readi, Hamdoon A. Mohammed

**Affiliations:** 1 Department of Pharmaceutics, Unaizah College of Pharmacy, Qassim University, Unaizah 51911, Saudi Arabia; 2 Department of Pharmacology and Toxicology, College of Pharmacy, Qassim University, Buraydah 51452, Saudi Arabia; 3 Department of Pharmaceutics, College of Pharmacy, Qassim University, Buraydah 51452, Saudi Arabia; 4 Department of Biochemistry, Faculty of Medicine, Al-Azhar University, Assiut 71524, Egypt; 5 Department of Clinical Biochemistry, Faculty of Medicine, Umm Al-Qura University, Makkah 21955, Saudi Arabia; 6 Department of Biochemistry, Faculty of Pharmacy, Al-Azhar University, Assiut 71524, Egypt; 7 Department of Medicinal Chemistry and Pharmacognosy, College of Pharmacy, Qassim University, Buraydah 51452, Saudi Arabia; 8 Department of Pharmacognosy and Medicinal Plants, Faculty of Pharmacy, Al-Azhar University, Cairo 11371, Egypt

**Keywords:** cinnamaldehyde (CA), SNEDDS, CA-SNEDDS, antimicrobial activity, antibiofilm, antioxidant, anti-inflammatory, wound healing, NAP-3, SOD, CAT

## Abstract

Cinnamaldehyde, the main phytoconstituent of the cinnamon oil, has been reported for its potential wound healing activity, associated to its antimicrobial and anti-inflammatory effects. In this study, we are reporting on the cinnamaldehyde-based self-nanoemulsifying drug delivery system (CA-SNEDDS), which was prepared and evaluated for its antimicrobial, antioxidant, anti-inflammatory, and wound healing potential using the rat third-degree skin injury model. The parameters, i.e., skin healing, proinflammatory, and oxidative/antioxidant markers, were evaluated after 3 weeks of treatment regimens with CA-SNEDDS. Twenty rats were divided randomly into negative control (untreated), SNEDDS control, silver sulfadiazine cream positive control (SS), and CA-SNEDDS groups. An aluminum cylinder (120 °C, 10-s duration) was used to induce 3rd-degree skin burns (1-inch square diameter each) on the rat’s dorsum. At the end of the experiment, skin biopsies were collected for biochemical analysis. The significantly reduced wound size in CA-SNEDDS compared to the negative group was observed. CA-SNEDDS-treated and SS-treated groups demonstrated significantly increased antioxidant biomarkers, i.e., superoxide dismutase (SOD) and catalase (CAT), and a significant reduction in the inflammatory marker, i.e., NAP-3, compared to the negative group. Compared to SNEDDS, CA-SNEDDS exhibited a substantial antimicrobial activity against all the tested organisms at the given dosage of 20 µL/disc. Among all the tested microorganisms, MRSA and *S. typhimurium* were the most susceptible bacteria, with an inhibition zone diameter (IZD) of 17.0 ± 0.3 mm and 19.0 ± 0.9 mm, respectively. CA-SNEDDS also exhibited strong antifungal activity against *C. albicans* and *A. niger*, with IZD of 35.0 ± 0.5 mm and 34.0 ± 0.5 mm, respectively. MIC and MBC of CA-SNEDDS for the tested bacteria ranged from 3.125 to 6.25 µL/mL and 6.25 to 12.5 µL/mL, respectively, while the MIC and MBC for *C. albicans* and *A. niger* were 1.56 µL/mL and 3.125 µL/mL, respectively. The MBIC and MBEC of CA-SNEDDS were also very significant for the tested bacteria and ranged from 6.25 to 12.5 µL/mL and 12.5 to 25.0 µL/mL, respectively, while the MBIC and MBEC for *C. albicans* and *A. niger* were 3.125 µL/mL and 6.25 µL/mL, respectively. Thus, the results indicated that CA-SNEDDS exhibited significant wound healing properties, which appeared to be attributed to the formulation’s antimicrobial, antioxidant, and anti-inflammatory effects.

## 1. Introduction

Cinnamaldehyde (CA, 3-phenyl-2-propenal), the phenylpropanoid volatile oil, is one of the major active constituents in several medicinal *Cinnamomum* species, e.g., C. verum, *C. zeylanicum*, *C. cassia*, and *C. osmophloeum* [1,2,3,4]. CA has been predominantly used as a flavoring agent in beverages, a fumigating agent, and has been reported for several medicinal purposes [5]. The anti-inflammatory effect of CA has been reported where CA was identified as an NF-κB inhibitor [6] and in reduction in Toll-like receptors 4, which activate the inflammatory response to forging molecules and microorganisms [5]. CA has also been shown to reduce proinflammatory cytokines TNF-α and IL-6 while increasing antioxidant response in rheumatoid arthritis patients’ peripheral blood mononuclear cells [7]. In addition, CA has also been reported for its antimicrobial and anticancer effects [8,9]. The compound has induced antimicrobial activity against microbial pathogens in foods [10] and microbial biofilms in wounds [11].

The wound healing potential of CA has also been investigated in several reports. For example, CA induced cell proliferation and angiogenesis through activating phosphatidylinositol 3-kinase, mitogen-activated protein kinase, and vascular endothelial growth factor expression in human umbilical vein endothelial cells [12]. At the same time, CA has increased wound closure in diabetic mice and exhibited proangiogenic potency in the Matrigel plug assay [12]. In addition, gelatin/chitosan and polyurethane cross-linkages incorporating CA demonstrated efficient application as a wound dressing due to the antimicrobial activity of CA [13,14]. Similarly, CA has been incorporated with several polymer dressings, e.g., β-cyclodextrin fibers [15], gellan/polyvinyl alcohol fibers [11], and collagen/polysaccharide hybrid scaffolds [16], to impart the antimicrobial property to these dressings. Additionally, CA has potential wound healing activity in *P. aeruginosa*-infected mouse skin wounds and reduces the level of nitric oxide, vascular endothelial growth factor, and IL-17 generation [17]. 

Wounds are a widespread problem and one of the leading causes of death. In addition, the skin, the largest organ in the human body, plays a critical role in human health as a barrier against microbial invasion and harmful radiation. Skin also plays an important role in regulating body temperature [18]. Injuries and burns are the most common causes of skin wounds that compromise skin integrity. This type of skin lesion is connected to severe infections, incapacity, and mortality in people who have it [19,20]. Several wound healing and skin protection procedures have been documented [21,22]. Skin wounds occur when the skin’s natural anatomical and functional condition is disrupted [23].

On the other hand, wound healing is a complicated process that restores wounded tissue to its original state [24]. Hemostasis, inflammation, proliferation, and modification of skin cells are all part of the skin healing process, which helps prevent complicated metabolic changes that impact bodily organ systems [25]. In contrast to wound incisions, tissue burns cause significant damage and require a complex healing network [24]. First-, second-, and third-degree burns are classified according to the depth of the burn wounds [26]. A first-degree burn is often red or gray, with no blisters and a normal capillary network, whereas a second-degree burn has blisters and partial-thickness dermal damage. Second- and third-degree burns are treated similarly [27]. Silver sulfadiazine (SS), an antibiotic, has been used to treat skin injuries. A recent meta-analysis indicated substantial effectiveness of SS in healing skin-burned rats faster than the control group after 3 weeks of treatment. Hence, SS is used as a positive control [28]. Compared to synthetic medications, several plants and their herbal derivatives are inexpensive to obtain and have modest medicinal potential with low toxicity [29,30,31]. Furthermore, improving the wound healing potential of a promising pure natural product, cinnamaldehyde (based on the literature), in a new formulation is an important study that may contribute to reducing wound/burn complications. 

A self-nanoemulsifying drug delivery system (SNEDDS) is used to encapsulate the insoluble lipophilic drug in a dissolved form with a size of 200 nm or less [32,33], in addition to improving solubility, augmenting absorption rate, providing increased enzymatic and chemical stability, and enhancing oral bioavailability [34,35]. The SNEDDS forms spontaneously from the mixture of oil, surfactant, co-surfactant, and an active drug compound in a homogenous fine oil-in-water nanoemulsion liquid form [36].

The current study evaluated the wound healing potential of CA as one of the most abundant volatile oils from the plant *C. cassia*. CA was formulated in a SNEDDS fabrication and evaluated for its potential wound healing activity and its closely related antimicrobial effect. To our knowledge, this is the first report concerning CA-SNEDDS wound healing activity and measuring the levels of the antioxidant and anti-inflammatory markers that support this effect.

## 2. Results and Discussion 

### 2.1. Physicochemical Characterization of SNEDDS and CA-SNEDDS

#### 2.1.1. Particle Size and Polydispersity Index

The average particle size and polydispersity index are essential metrics in nanoparticle characterization because they predict the stability of these nanosamples, particularly CA-SNEDDS. The average particle size of SNEDDS was 31.59 nm, while the polydispersity index (PDI) obtained was 0.651, whereas CA-SNEDDS had an average particle size of 38.63 nm with a PDI of 0.431. Both size and PDI showed that SNEDDS and CA-SNEDDS are good nanoformulations as their size is less than 200 nm, which improves the drug (CA) solubility and absorption by increasing the interfacial area [37]. Figures S1 and S2 in the Supplemental File exhibit the findings from the Malvern Instruments report, demonstrating that most of the particles were distributed homogeneously and reflecting the formulation’s quality.

#### 2.1.2. Surface Charge Analysis

A stable colloidal dispersion has a zeta potential between −10 and +10 mV, but zeta potential values of nanodispersions larger than 30 mV and less than −30 mV are regarded as strongly cationic and anionic, respectively [33]. The results obtained from the Malvern Instruments report (Supplementary File, Figures S3 and S4) stated that the zeta potential value of SNEDDS obtained was −9.70 mV, whereas, for CA-SNEDDS, it was −9.90 mV, which is considered neutral and stable as the sample is monodispersed (single peak) and corresponds to a single component zeta potential value [33].

#### 2.1.3. Fourier-Transform Infrared Spectroscopy (FT-IR)-Based Computability Analysis of CA-SNEDDS

**Figure 1:**
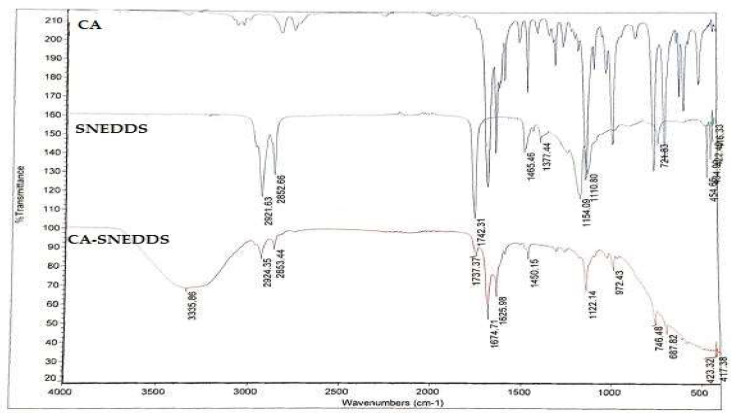
FT-IR analysis of CA, SNEDDS, CA-SNEDDS. Note: CA = pure cinnamaldehyde; SNEDDS = SNEDDS without CA; CA-SNEDDS = SNEDDS with CA.

FT-IR is among the most efficient approaches used for estimating the chemical stability of the components following their formulation in the SNEDDS formula [38]. Therefore, FT-IR analysis was conducted to check the computability of the CA-SNEDDS formulation and exclude any possibility of a chemical reaction between cinnamaldehyde and the other ingredients involved in the preparation of the SNEDDS. The results in Figure 1 (CA) revealed the presence of a stretch at 1667 cm^−1^ corresponding to the carbonyl group C=O of the cinnamaldehyde. The spectra also showed stretches at 1449–1624 cm^−1^ assigned to the alkene group (C=C bond); 2813 cm^−1^ related to the =C–H bond; 2742 cm^−1^ related to the C–H bond of the carbonyl group; and 3061 cm^−1^ (m) related to the aromatic C–H bond, which were all consistent with the published FT-IR data of cinnamaldehyde [4,39]. Figure 1 (CA-SNEDDS) shows the same peaks of the cinnamaldehyde plus the peaks of the SNEDDS vehicle, which indicate the absence of chemical interaction between CA and the other ingredients of the SNEDDS vehicle. 

### 2.2. Antimicrobial Activity of CA-SNEDDS

In this study, the selection of the test organisms is based on the fact that these are the most prevalent human pathogenic organisms responsible for wound infections in human beings [40,41].

#### 2.2.1. Preliminary Antimicrobial Activity 

**Figure 2:**
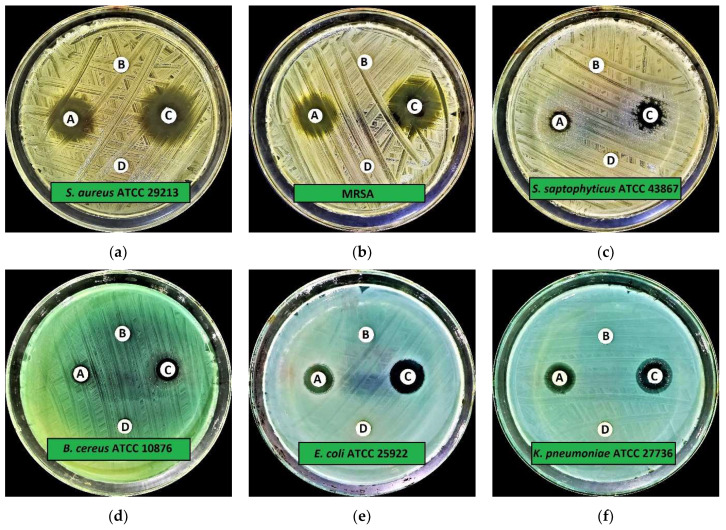

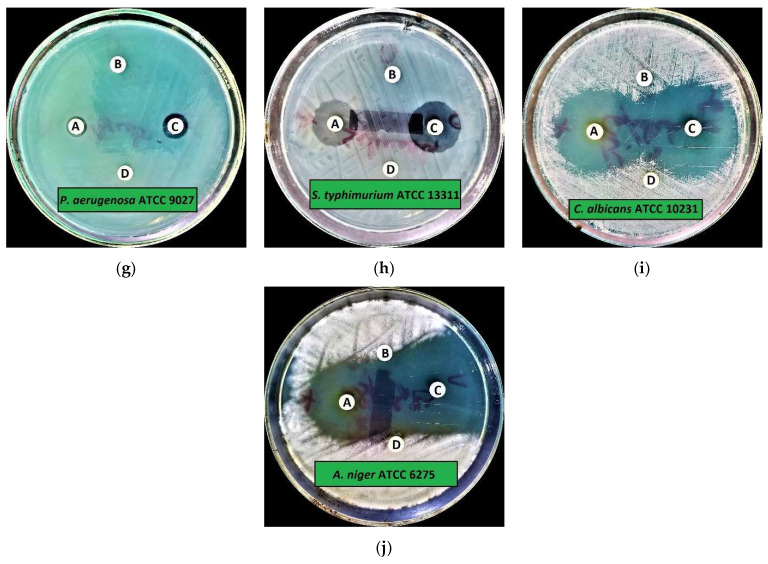
Preliminary antimicrobial activity of CA-SNEDDS: (**a**) *S. aureus* ATCC 29213; (**b**) MRSA; (**c**) *S. saptophyticus* ATCC 43867; (**d**) *B. cereus* ATCC 10876; (**e**) *E. coli* ATCC 25922; (**f**) *K. pneumoniae* ATCC 27736; (**g**) *P. aeruginosa* ATCC 9027; (**h**) *S. typhimurium* ATCC 13311; (**i**) *C. albicans* ATCC 10231; (**j**) *A. niger* ATCC 6275. Note: A = CA-SNEDDS; B = SNEDDS; C = Positive control; D = Negative control.

**Figure 3:**
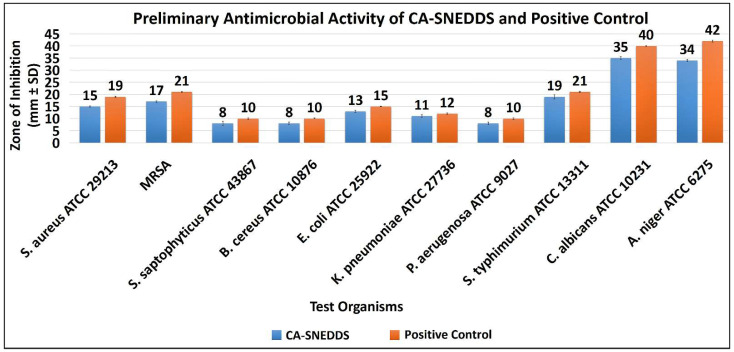
Preliminary antimicrobial activity of CA-SNEDDS.

The preliminary antimicrobial activity demonstrated that CA-SNEDDS exhibited substantial antimicrobial activity against all the tested organisms, whereas SNEDDS did not show antimicrobial activity against the tested organisms at the given dosage of 20 µL/disc (Table 1 and Figure 2 and Figure 3). The results further demonstrated that the positive control (5% *v/v* cinnamaldehyde in DMSO) had significantly inhibited the growth of all the tested organisms. In contrast, the negative control (DMSO) did not affect the growth of tested organisms at the given dosage of 20 µL/disc (Table 1 and Figure 2 and Figure 3).

The antibacterial activity of CA-SNEDDS revealed that MRSA and *S. typhimurium* ATCC 13311 were the most susceptible tested bacteria, with IZD (mm ± standard deviation (SD)) of 17.0 ± 0.3 mm and 19.0 ± 0.9 mm, respectively, at the given dosage of 20 µL/disc. In addition, the results further revealed that *S. saptophyticus* ATCC 43867, *B. cereus* ATCC 10876, and *P. aeruginosa* ATCC 9027 were the least susceptible test bacteria, with IZD of 8.0 ± 0.9 mm, 8.0 ± 0.5 mm, and 8.0 ± 0.5 mm, respectively, at the given dosage of 20 µL/disc (Table 1 and Figure 2 and Figure 3). 

The antifungal activity of CA-SNEDDS demonstrated that both *C. albicans* ATCC 10231 and *A. niger* ATCC 6275 were highly susceptible to it, with IZD of 35.0 ± 0.5 mm and 34.0 ± 0.5 mm, respectively, at the given dosage of 20 µL/disc (Table 1 and Figure 2 and Figure 3).

#### 2.2.2. Minimum Inhibitory Concentration (MIC), Minimum Biocidal Concentration (MBC), Minimum Biofilm Inhibitory Concentration (MBIC), and Minimum Biofilm Eradication Concentration (MBEC)

The MIC and MBC results of CA-SNEDDS revealed that the MIC values for the tested bacteria ranged from 3.125 to 6.25 µL/mL, while MBC values ranged from 6.25 to 12.5 µL/mL (Table 2). The MIC and MBC results for the tested fungi demonstrated that *C. albicans* ATCC 10231 and *A. niger* ATCC 6275 both exhibited MIC and MBC values of 1.56 µL/mL and 3.125 µL/mL, respectively (Table 2). 

The MBIC and MBEC results of CA-SNEDDS revealed that the MBIC values for the tested bacteria ranged from 6.25 to 12.5 µL/mL, whereas the MBEC values ranged from 12.5 to 25.0 µL/mL. The MBIC and MBEC results for the tested fungi demonstrated that *C. albicans* ATCC 10231 and *A. niger* ATCC 6275 both exhibited MBIC and MBEC values of 3.125 µL/mL and 6.25 µL/mL, respectively (Table 2).

#### 2.2.3. Statistical Analysis

There is a statistically significant difference (*p* < 0.05) between the antimicrobial activity of CA-SNEDDS among the tested organisms as determined by one-way ANOVA; F (9, 20) = 975.080, *p* = 0.000 (Table S1).

CA-SNEDDS displayed significant antimicrobial activity against the tested organisms, as shown by the overall antimicrobial results. This activity seems to be attributable to CA since the absence of CA in the SNEDDS formulation rendered it inactive against microorganisms. In addition, several researchers have reported that CA is a potent antimicrobial agent against Gram-positive and Gram-negative bacteria and fungi [42,43,44,45,46,47,48]. 

Doylea and Stephens have reported that CA has substantial antimicrobial and antibiofilm activity against various human pathogens, including Gram-positive and Gram-negative bacteria and fungi. This supports our study showing that CA exhibited substantial antimicrobial activity against Gram-positive and Gram-negative bacteria and fungi [42]. Liefferinge et al. found that CA exhibited strong antibacterial activity against *E. coli*. This result corroborates our findings, which indicates that CA has significant antibacterial efficacy against *E. coli* [43]. He et al. reported that CA exhibited significant antimicrobial activity against *S. mutans* with MIC and MBC values of 1000 and 2000 μg/mL, respectively. They also reported that CA decreased the biofilm biomass and metabolism of *S. mutans* at sub-MIC concentrations. This study also corroborates our findings that CA has significant antimicrobial activity against several human pathogens [44]. Cox et al. incorporated the CA-based preparations into mussel-inspired and natural plant polyphenol coatings as part of a single-step fabrication process. They showed that the coatings containing polydopamine-cinnamaldehyde, polyethyleneimine-cinnamaldehyde, and tannic acid-cinnamaldehyde had potent antibacterial properties against *E. coli* and *S. aureus*. This investigation confirms that a CA-based preparation (CA-SNEDDS) exhibits significant antimicrobial activity against several human pathogens, such as *E. coli* and *S. aureus* [45]. Mousavi et al. showed that CA has significant antimicrobial activity against *E. coli*, which confirms our investigation, demonstrating that CA has high antibacterial activity against *E. coli* with MIC and MBC values of 3.125 µL/mL and 6.25 µL/mL, respectively [46]. Firmino et al. evaluated the antibiofilm activity of CA against several pathogens. They observed that the biofilm biomasses were decreased by up to 99.9%, and *S. pyogenes, P. aeruginosa,* and *E. coli* biofilms were very sensitive to all the concentrations used. This investigation validates our findings that CA inhibits the biofilms of different pathogens, including *P. aeruginosa* and *E. coli* [47]. Gill et al. found that CA is antibacterial against Gram-positive and Gram-negative bacteria. At 20 °C, 30 mM of CA was bactericidal in broth medium. This investigation corroborates our findings that CA has significant antibacterial properties against various human pathogens, including Gram-positive and Gram-negative bacteria and fungi [48]. 

### 2.3. In Vitro Antioxidant Activity of CA and CA-SNEDDS

Two in vitro methods were used to measure the antioxidant activity of the CA and CA-SNEDDS. The methods give an indication of the reducing capacity (total antioxidant capacity, TAC) and the 2,2-diphenyl-1-picrylhydrazyl (DPPH)-free radical scavenging activity (DPPH-SA) of the CA oil and CA-SNEDDS preparation. The in vitro antioxidant activity of CA has been reported previously, in which results have indicated the remarkable antioxidant activity of CA [49,50]. In the current study, we compared the in vitro antioxidant effect of CA and CA-SNEDDS to demonstrate whether CA antioxidant capacity was affected by the formulation of the compound in the form of CA-SNEDDS or not. The results shown in Table 3 indicated the marked in vitro antioxidant activity of CA and CA-SNEDDS. They exhibited TAC values of 18.16 and 17.12 mg Trolox equivalent and DPPH-SA values of 6.60 and 6.57 mg Trolox equivalent, respectively. The results in Table 3 also revealed slightly better free radical scavenging (DPPH-SA) and reducing power for the CA compared to the CA-SNEDDS. However, no significant differences were recorded, indicating that the antioxidant capacity of CA was maintained in the CA-SNEDDS and that other formulation ingredients did not affect CA’s antioxidant activity.

### 2.4. Wound Healing Activity of CA-SNEDDS

During the wound recovery stage, increased circulatory flow plays a vital role in the progression of angiogenesis and controlling inflammation and the spread of infection to other areas. Various animal models are employed for evaluating the skin healing process, among which rodents (rats and mice) are more commonly employed due to their faster reduction in the burned zone when compared to the epithelization rates in humans. In addition, the rat model has its limitations compared to humans, owing to their loose skin. However, rats are the most desired model due to their genetic and behavioral similarity, economic viability, and fewer constraints during experimentation [51,52]. The current study evaluated the wound healing potential of 5% CA-SNEDDS in the 3rd-degree burn rat model by measuring pro/antioxidant and inflammatory levels.

Silver sulfadiazine (SS) is a commercially available product used for burn wound healing and is commonly used as a standard wound healing agent (positive control) in the literature [50]. SS has strong antimicrobial activity against all microorganisms, including yeast, by destroying their respiratory pathways [51]. The antibacterial action of silver sulfadiazine, which benefits the wound bed, is one of the drug’s favorable effects. SS also reduces metalloproteinases to levels that encourage wound healing [52]. Additionally, silver formulations contribute to the modification or compression of inflammatory processes in wounds and to the facilitation of the initial stages of wound healing. Therefore, we have used SS as a positive control in the current study. 

**Figure 4:**
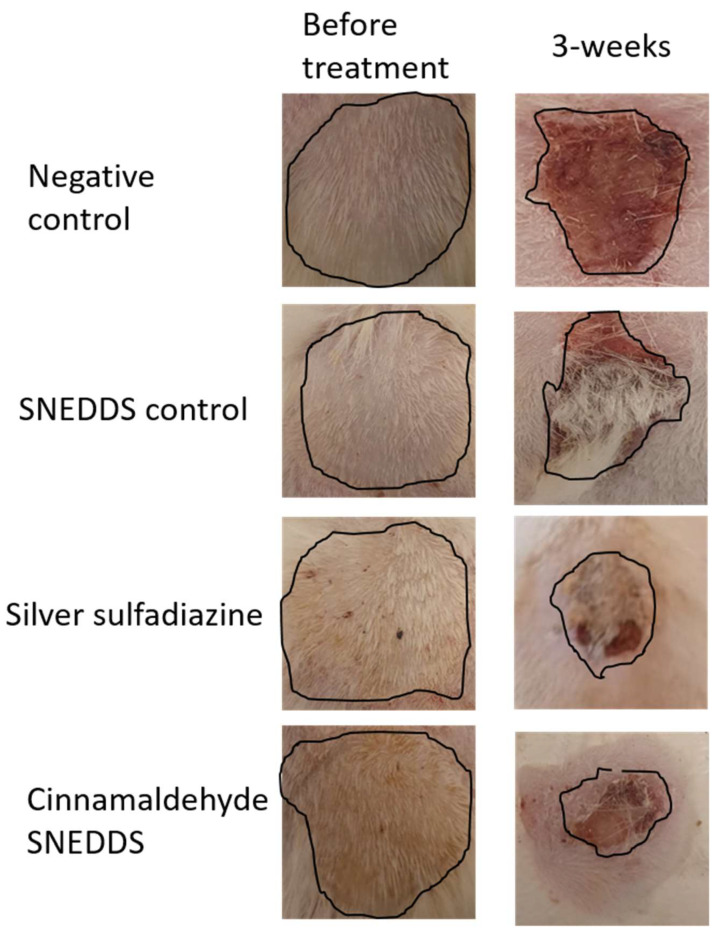
Morphological appearances of the negative, SNEDDS control, silver sulfadiazine, and cinnamaldehyde SNEDDS groups at the time of skin burn induction and the end of the experimental procedure. Image markings in black indicate the wound area for all the groups before and after treatment.

Treating wounds with CA-SNEDDS led to significant healing when compared to the negative group. After the burn induction, the burned rat skin appeared white, while 3 weeks after the experimental treatment procedure, the CA-SNEDDS and SS groups revealed a reduced wound zone compared to the negative or SNEDDS control groups, which had both edema formation and decreased wound healing (Figure 4). 

**Figure 5:**
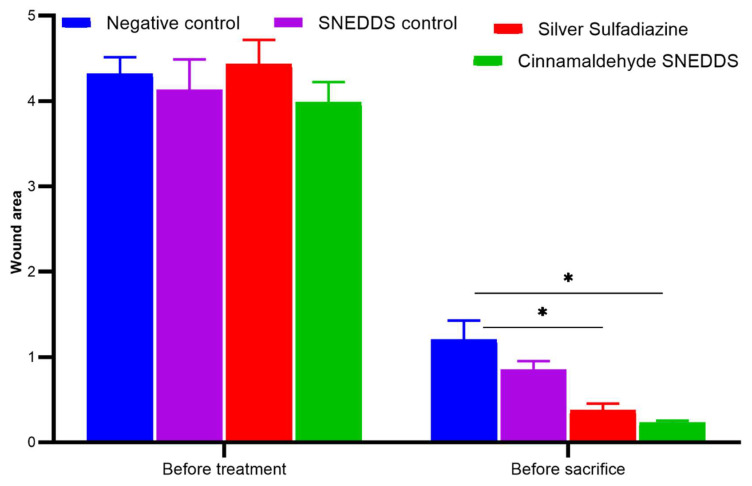
Wound size on day 0 of skin burn induction and after 3 weeks of treatment. Values are denoted as mean ± SEM, N = 5 rats per group. Statistical significance was found using two-way ANOVA, followed by a post hoc test in GraphPad Prism 8.0.2. * *p* < 0.05 using Tukey’s multi-group comparisons.

The wound zone was calculated using the ImageJ program. On day 0, the wound areas measured for all the groups were not statistically different, while at the end of the experiment, after 3 weeks, the treated groups, SS and CA-SNEDDS, were significantly different, while SNEDDS control did not differ significantly when compared to the untreated group (*p* < 0.05) (Figure 5).

### 2.5. Role of Oxidative/Antioxidant Markers in Skin Wound Healing

**Figure 6:**
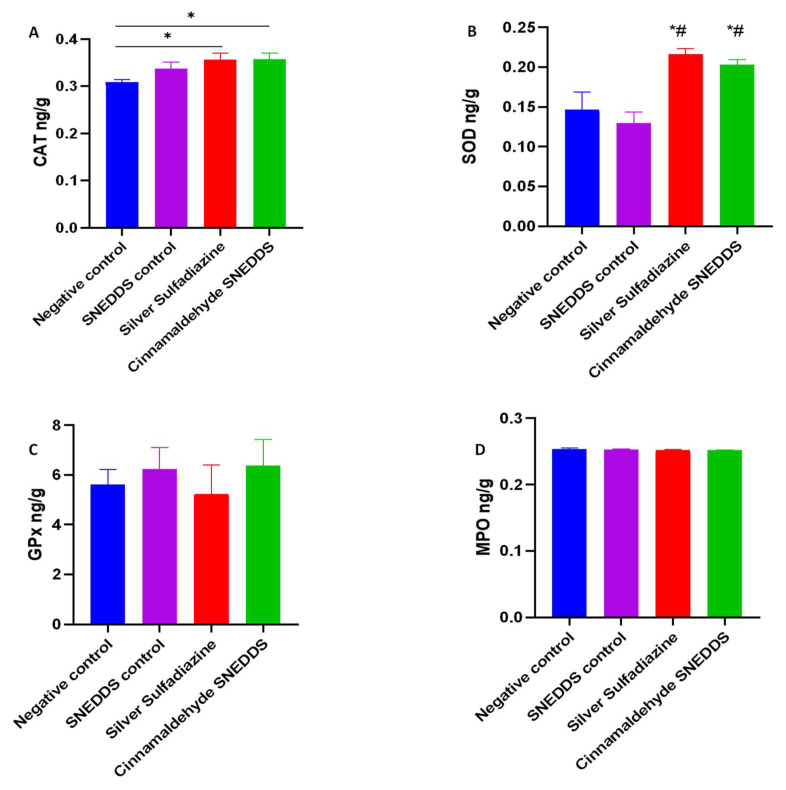
Effect of cinnamaldehyde on antioxidant and oxidant levels in the model of skin burn rats. Values are denoted as mean ± SEM, N = 5 rats per group. Statistical significance was found using one-way ANOVA, followed by a post hoc test in GraphPad Prism 8.0.2. (**A**) Catalase (CAT), (**B**) superoxide dismutase (SOD), (**C**) glutathione peroxidase (GPx), and (**D**) malonaldehyde peroxide (MPO). * *p* < 0.05 vs. negative control and # *p* < 0.01 vs. SNEDDS control using Tukey’s multi-group comparisons.

An antioxidant relationship during the wound recovery process has been demonstrated previously [53,54] in reducing inflammation and accelerating recovery of the injured zone [55]. The CAT and SOD enzymatic activities were significantly increased for SS and CA-SNEDDS (*p* < 0.05) groups, while the SNEDDS control remained comparable to the negative control group (Figure 6A,B). The GPx was found to be insignificant among all the groups (Figure 6A). In a recent thermal skin burn injury study, the systemic levels of MPO increased on day one and gradually decreased over 28 days [56]. In our study, the oxidative stress marker MPO at the tissue level was comparable among all the groups after 3 weeks of treatment (Figure 6D). The increased enzymatic activities of antioxidants CAT and SOD in CA-treated groups are in accordance with their wound healing potency [57]. 

Inflammatory cells such neutrophils and macrophages are recruited during the early days of skin burn [58,59] and play a role in secreting cytokines and reactive oxygen species (ROS), contributing to the dual role of protection against infectious organisms and delaying the healing process [60]. Control of immune cells and cytokines is required during the initial days of injury to continue the normal wound healing process [61]. As part of this process, abundant antioxidants control the induced oxidative stress through increased levels of SOD, CAT, and GPx [62]. 

### 2.6. Role of Pro/Anti-Inflammatory Markers in Skin Wound Healing

Inflammation plays a primary role in the wound progression and healing processes. At the same time, the inflammatory mediators produced by the infiltrated inflammatory cells are increased after injury and decrease gradually with the progress of healing processes. CA is known to play a key role in wound healing, as demonstrated during a clinical trial in which 2% cinnamon ointment on episiotomy incisions not only had analgesic properties but also contributed to significant wound healing [63]. In the study, cinnamon ointment rather than CA was used even though CA, along with eugenol and linalool, are known to be major components [64]. In addition, systemic administration of CA through the i.p. route in mice recently demonstrated significant wound healing potential, indicating the plausible use of multiple routes of administration [12]. CA has reduced NO and proinflammatory cytokine generation upon LPS stimuli [65,66,67,68]. In another study, CA reduced inflammation by reducing IL-17, VEGF, and NO in *P. aeruginosa*-infected mice [17].

Neutrophil recruitment at skin burns is known to occur [69,70,71]. MPO is a heme-containing enzyme connected to its bi-dimers by disulfide bonds [72]. MPO is stored in neutral granules of neutrophils and is not released until the neutrophil is activated or degranulated to achieve its corresponding physiological function [73]. Systemic neutrophil sequestration demonstrated increased vascular damage and other complications in skin-burned patients [74]. Preventing neutrophil adhesion to the vascular wall is a robust approach to the reduction in pulmonary neutrophil sequestration as well as vascular permeability [75,76].

**Figure 7:**
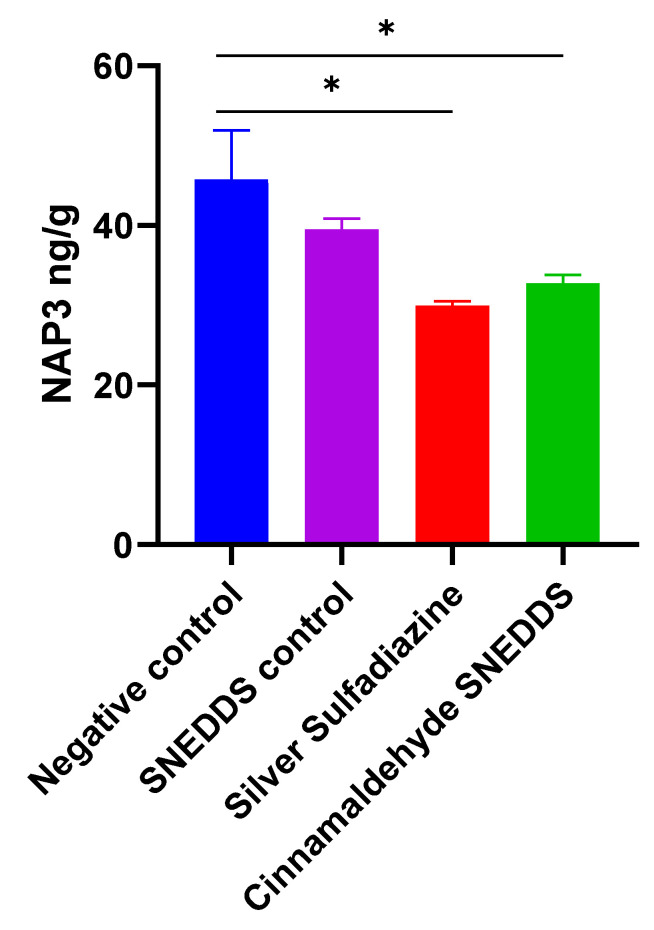
Effect of cinnamaldehyde on the inflammatory marker in skin burn rat model. Values are denoted as mean ± SEM, N = 5 rats per group. Statistical significance was found using one-way ANOVA, followed by a post hoc test in GraphPad Prism 8.0.2. NAP3: Neutrophil-activating protein 3. * *p* < 0.05 vs. negative control using Tukey’s multi-group comparisons.

In the current study, NAP3 was significantly reduced in CA-SNEDDS and SS, while SNEDDS control remained comparable to the negative group (*p* < 0.05) after 3 weeks of treatment, indicating a decreased inflammatory profile (Figure 7). 

## 3. Materials and Methods

### 3.1. Materials

CA (>98% purity) was procured in the form of active pharmaceutical ingredient (API) from AdooQ BioScience, Irvine, CA, USA. This registered vendor already checked and claimed the purity; hence, there is no need to recheck the purity of this procured CA.

### 3.2. Formulation of SNEDDS and CA-SNEDDS

**Figure 8:**
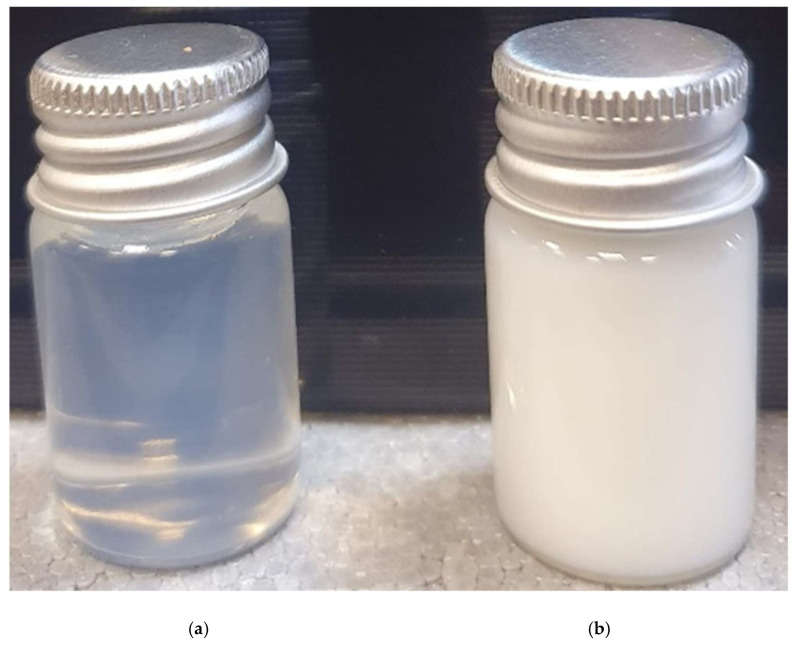
(**a**) SNEDDS; (**b**) CA-SNEDDS.

The method for producing SNEDDS from a previously published article was used [33]. SNEDDS formed as a clear and transparent solution with minimal opalescence (bluish color), no foam, no oil separation, and no particle sedimentation, demonstrating good nanoformulation qualities [33]. The oil phase dispersion comprised coconut oil and Tween 80, and the SNEDDS was loaded with CA (>98% pure) (5% *v/v*) (1:5). The oil phase dispersion was then melted by heating it over its melting point to guarantee breakage of lipid condition, thereby avoiding the lipid memory effect. Dimethyl sulfoxide (2% *v/v*) was used to form the aqueous phase dispersion, and distilled water was added to make the volume 100 mL. After that, CA was added to the SNEDDS formulation and stirred for 72 h at room temperature under constant observation. The nanoformulation was filtered and promptly put into clean glass vials after CA-SNEDDS was synthesized (Figure 8).

### 3.3. Characterization of CA-SNEDDS Formulation

CA-SNEDDS formulation was characterized in terms of its particle size, polydispersity index (PDI), zeta potential, and FT-IR spectroscopic analysis.

#### 3.3.1. Particle Size and Polydispersity Index

Dynamic light scattering (DLS) integrated into the Zetasizer Nano ZS (Malvern Instruments, Malvern, Germany) was used to analyze the average particle size and the size distribution (polydispersity index, PDI) of CA-SNEDDS. The measurement was conducted at 25 °C. 

#### 3.3.2. Surface Charge Analysis

A Zetasizer Nano ZS (Malvern Instruments, Malvern, Germany) was used to obtain the zeta potential of the CA-SNEDDS formulation. The zeta potential, or surface charge, measures the magnitude of charges between particles. The zeta potential is used to anticipate the sample’s long-term durability since it indirectly evaluates the thickness of the nanoparticles’ diffusion layer.

#### 3.3.3. FT-IR Analysis

FT-IR spectrometric analysis was conducted to detect any incompatibilities in the CA-SNEDDS formulation [77]. It was measured with a Bruker Tensor 27 FT-IR spectrophotometer (Varian Company model: 640-IR, Mulgrave, Victoria, Australia). The FT-IR spectra were recorded, and the absorption peaks were observed at 400–4000 cm^−1^.

### 3.4. Antimicrobial Activity of CA-SNEDDS

#### 3.4.1. Test Organisms

Four Gram-positive human pathogenic bacteria: *S. aureus* ATCC 29213, MRSA (clinical isolate), *S. saprophyticus* ATCC 43867, *B. cereus* ATCC 10876; four Gram-negative human pathogenic bacteria: *E. coli* ATCC 25922, *K. pneumoniae* ATCC 27736, *P. aeruginosa* ATCC 9027, *S. typhimurium* ATCC 13311; and two human pathogenic fungal strains: *C. albicans* ATCC 10231 and *A. niger* ATCC 6275, were used as test organisms. All the test organisms (ATCC) were procured from Microbiologics, Biotechnology Company, St Cloud, MN, USA, while one clinical isolate (MRSA) was collected from the microbiology department of King Saud Hospital, Unaizah, Saudi Arabia. 

#### 3.4.2. Preliminary Antimicrobial Activity

The preliminary antimicrobial activity of CA-SNEDDS and control samples was examined by the disc diffusion technique [78,79,80]. Modified Mueller–Hinton agar (MMHA) and potato dextrose agar (PDA) media were utilized to determine the antibacterial and antifungal activities, respectively. The MMHA and PDA were prepared by following the manufacturer’s instructions and a previous study [78]. Sterile discs with a 6 mm diameter were employed in this investigation. Each disc was impregnated with its respective sample with 20 µL/disc. Before inoculating impregnated discs on the surface of the test media, the impregnated discs were sterilized by ultra-violet (UV) light for 20 min.

The 5% (*v/v*) CA in DMSO and pure DMSO were used as a positive and negative control, respectively. Each organism’s inoculum was prepared in sterile tryptic soy broth (TSB), and the turbidity of each suspension was adjusted to equal 0.5 MacFarland standard at OD_600_ (0.08–0.12). Following that, 100 µL of each adjusted inoculum was dispensed individually and separately onto the surface of its respective test media plates, and then suspensions were evenly spread using sterile cotton swabs. After that, the prepared discs were placed on the surface of inoculated media plates. All the plates were incubated at 35 ± 2 °C for 24 h for bacteria and 48 h for fungi. After incubation, the diameters of inhibitory zones were measured on a millimeter (mm) scale. Each test was performed in triplicate. The results are expressed in mm ± SD. 

#### 3.4.3. MIC and MBC

MIC was determined by the resazurin-based broth microdilution method, while MBC was performed following the standard spot inoculation method [78,79,81,82]. The stock solution of CA-SNEDDS was prepared in DMSO with a 100 µL/mL concentration, and then 200 µL stock solution was dispensed in each well of column 1, while columns 2–10 contained 100 µL of TSB only. Column 11 had 200 µL of standardized inoculum suspensions as a negative control (NC), while column 12 consisted of 200 µL of sterile TSB as sterility control (SC). A two-fold serial dilution of CA-SNEDDS was prepared by mixing and transferring the CA-SNEDDS solution from columns 1 to 10 with a multi-channel pipette, yielding 100 µL/well. The tested concentrations of the CA-SNEDDS achieved through a two-fold serial dilution from columns 1–10 were 100–0.195 µL/mL. 

The inocula of each test bacteria were prepared in TSB, following the CLSI guidelines, where the OD_600_ value (0.08–0.12) was adjusted, resulting in ~1 × 10^8^ CFU/mL. Then, adjusted inocula were further diluted by 1:100 in TSB, resulting in ~1 × 10^6^ CFU/mL. In contrast, the inocula of test fungi were prepared in potato dextrose broth (PDB) following the CLSI guidelines, where the OD_600_ value (0.08–0.12) was adjusted, and the resulting stock suspension contained 1 × 10^6^ to 5 × 10^6^ CFU/mL for yeast and 4 × 10^5^ to 5 × 10^6^ CFU/mL for mold. A working yeast suspension was prepared by a 1:100 dilution followed by a 1:20 dilution of the stock suspension with PDB, resulting in 5.0 × 10^2^ to 2.5 × 10^3^ cells/mL, while a working mold suspension was prepared by a 1:50 dilution of the stock suspension with PDB, resulting in 0.8 × 10^4^ to 1 × 10^5^ cells/mL. 

The 100 µL of adjusted microbial inocula was dispensed in all the wells of columns 1–10, resulting in ~5 × 10^5^ CFU/mL for bacteria and ~2.5 × 10^2^ to 1.25 × 10^3^ CFU/mL for *C. albicans*, and 0.4 × 10^4^ to 5 × 10^4^ CFU/mL for *A. niger*. Thus, the resulting concentrations of CA-SNEDDS from columns 1–10 were 50–0.098 µL/mL. The time to prepare and dispense the OD-adjusted microbial inocula did not exceed 15 min. All inoculated plates were incubated at 35 ± 2 °C for 24 h for bacteria and 48 h for fungi. Following the incubation, the 30 µL of sterile resazurin (0.015%, *w/v*) solution was dispensed in each well and re-incubated for 1–2 h to observe color change. Following incubation, the columns that remained blue in color were recorded as above the MIC. 

MBC was determined by directly plating the contents of wells with concentrations above the MIC on sterile tryptic soy agar (TSA) plates for bacteria and potato dextrose agar (PDA) plates for fungi. The lowest concentration of CA-SNEDDS that did not produce isolated colonies of the tested organisms on the inoculated agar plates was considered the MBC.

#### 3.4.4. MBIC and MBEC

MBIC is the lowest antimicrobial agent concentration preventing the tested organism’s biofilm formation. MBIC was examined against all the test organisms. A 96-well microtiter plate was used to evaluate the antibiofilm activity of CA-SNEDDS [78]. The inocula of the test organisms were prepared in TSB for bacteria and PDB for fungi, equal to 0.5 MacFarland standard (1–2 × 10^8^ CFU/mL for bacteria, 1 × 10^6^ to 5 × 10^6^ CFU/mL for yeast, and 4 × 10^5^ to 5 × 10^6^ CFU/mL for mold). An aliquot of 100 μL from the adjusted inocula was dispensed into each test well of a 96-well plate. Then, 100 μL of different concentrations of CA-SNEDDS was dispensed into test wells. Thus, the final concentrations for MBIC assessment were MIC, 2 × MIC, and 4 × MIC. The blank control (BC) wells contained 200 μL sterile TSB/PDB. The plates were incubated at 35 ± 2 °C for 24 h for bacteria and 48 h for fungi. After incubation, the supernatants from each well were decanted gently by reversing the plates on a tissue paper bed/or through a micropipette. The plates were dried in air for 30 min, stained with 0.1% (*w/v*) crystal violet at room temperature for 30 min, and then washed thrice with distilled water. Subsequently, the crystal violet was solubilized by adding 200 µL of 95% *v/v* ethanol into each test well. The absorbance was recorded in a microplate reader (xMark™ Microplate Absorbance Spectrophotometer, Bio-Rad, Hercules, CA, USA) at 650 nm. The lowest concentration of CA-SNEDDS at which the absorbance equals or falls below the BC is recorded as MBIC. Each test was performed in triplicate. The mean of three independent tests was taken. The results are expressed in µL/mL. 

MBEC is the minimum antimicrobial agent concentration that eradicates the test organism’s biofilm [78]. A 200 μL inoculum equal to 0.5 MacFarland standard (1–2 × 10^8^ CFU/mL for bacteria, 1 × 10^6^ to 5 × 10^6^ CFU/mL for yeast, and 4 × 10^5^ to 5 × 10^6^ CFU/mL for mold) of each test organism was inoculated into each test well of a flat-bottom 96-well microtiter plate. The plates were incubated at 35 ± 2 °C for 48 for bacteria and 72 h for fungi for biofilm formation. After forming the biofilms, the contents of the test wells were discarded by inverting the plates over a tissue paper bed/or through a pipette to remove non-adherent cells. The various CA-SNEDDS concentrations, MIC, 2 × MIC, and 4 × MIC, were added to different test wells (200 μL/well). The inoculated plates were re-incubated at 35 °C for 24 h. After incubation, the contents of each test well were discarded by inverting the plates on a tissue paper bed/or through a pipette. The plates were dried in air for 30 min, and then 200 μL of sterile TSB/PDB was dispensed in each test well. After that, 30 µL of 0.015% *w/v* resazurin dye was added to each test well. The plates were re-incubated for 1–2 h. After re-incubation, the MBEC values were recorded by observing the color change from blue to pink. The column with no color change (blue resazurin color stayed intact) was scored as MBEC.

### 3.5. In Vitro Antioxidant Assay

The in vitro antioxidant activity of CA and CA-SNEDDS was measured by two different assays, i.e., DPPH free radical scavenging activity (DPPH-SA) and total antioxidant capacity (TAC), using the methods described in the literature [83,84]. In the DPPH-SA assay, 1 mL of the DPPH working solution (prepared by dissolving 6 mg of the DPPH in 50 mL of methanol) was thoroughly mixed with 1 mL of CA or CA-SNEDDS in methanol (containing 0.5 mg of CA). The mixture was kept in the dark for 30 min, and the DPPH color reduction was measured at 517 nm using a spectrophotometer. A calibration curve for the DPPH–Trolox interaction was plotted, whereas the DPPH-SA of the samples was measured as Trolox equivalent per mg of the samples.

In the TAC assay, 2 mL of the freshly prepared molybdate reagent (consisting of sulfuric acid (0.6 M), ammonium molybdate (4 mM), and sodium phosphate buffer (28 mM)) was mixed with 200 µL of CA or CA-SNEDDS in methanol (containing 0.5 mg of CA). A blank test was prepared by mixing 2 mL of the molybdate reagent with 200 µL of distilled water. The tubes were heated to 85 °C for 30 min before being allowed to cool to room temperature. The developed blue color was measured spectrophotometrically at 695 nm using the blank reading as an auto-zero point. The TAC of the compounds was calculated using the Trolox standard calibration curve. 

### 3.6. Wound Healing Experiment

#### 3.6.1. Animal Groups

Twenty 3-month old Sprague Dawley female rats (200 ± 50 g) were individually maintained in a cage under 25 ± 2 °C, 65% humidity, 12:12 light–dark cycle. The wound healing study was conducted following the Institutional Animal Ethics Committee (Registration no. 210406). The animal groups involved were the negative control group, SNEDDS control (treated with SNEDDS vehicle), positive control (1% silver sulfadiazine, SS), and cinnamaldehyde self-nanoemulsifying drug delivery system (CA-SNEDDS). 

#### 3.6.2. Skin Burn Induction

According to our previously published article, the skin burn induction method was followed [82]. Briefly, the animals were anesthetized using ketamine-xylazine, and the rat’s dorsum was shaved at a 45° angle to minimize skin injury. Using a pre-heated aluminum cylinder (86 g weight) at 110 °C, 3rd-degree burns of 1-inch square diameter were induced on the shaved area. Following the induction, all rats were injected i.p. with 0.9% normal saline (10 mL/kg) followed by twice-daily topical application of SNEDDS control or 1% SS cream or 5% CA-SNEDDS on the wound area while the negative group’s wound area remained untreated. At the end of the experimental time frame, biopsies (1 × 1 cm diameter) obtained from the euthanized animals were homogenized, followed by isolation of supernatant for biochemistry or fixed in 3.7% formalin for paraffin embedding. 

#### 3.6.3. Determination of Oxidants and Antioxidants

CAT, SOD, and MPO levels were determined in tissue homogenates using ELISA kits following the manufacturer’s protocol, and absorbance was read at 450 nm. The concentration was determined using a standard curve. 

#### 3.6.4. Determination of Inflammatory Marker in Skin Wound Tissue

According to the manufacturer’s instructions, the proinflammatory cytokine neutrophil-activating protein 3 (NAP3) was assayed in homogenated skin wound tissue with an ELISA kit (Cloud Clone Corp, Katy, TX, USA). The microplates were measured with a 450 nm filter by a microplate reader.

### 3.7. Statistical Analysis

Values were expressed as the mean ± standard error of the mean (SEM) (n = 5). Variation among groups was evaluated using one-way or two-way ANOVA and Tukey’s multi-group comparison post hoc test in GraphPad Prism 8.0.2, San Diego, CA, USA. *p* < 0.05 was determined to be statistically significant [85]. The preliminary antimicrobial activity of Mgl was statistically analyzed using the one-way ANOVA statistical test to determine statistical differences among the means of tested organisms. The post hoc test (Tukey’s method) was performed to assess the significance of interactions among the means of groups, where *p* = 0.05 was considered statistically significant. The SPSS software, version 20.0 (IBM, New York, NY, USA), was used to conduct the statistical analysis. 

## 4. Conclusions

**Figure 9:**
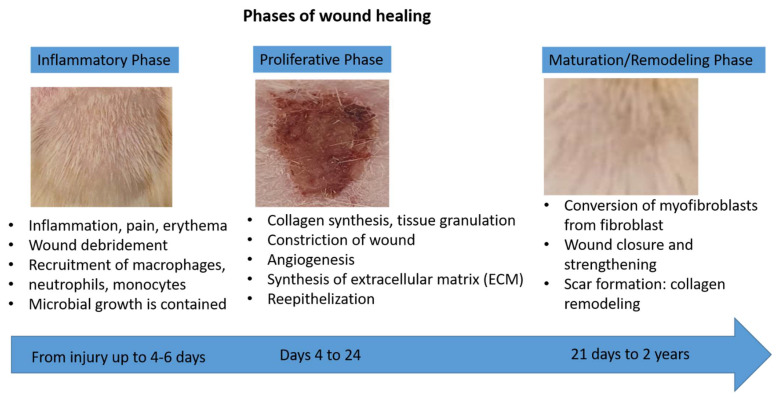
Phases of wound healing.

Based on the findings of this study, treatment with CA-SNEDDS demonstrates wound healing properties and exhibits substantial antimicrobial and antibiofilm activity against a broad spectrum of microorganisms. The study also proves that CA-SNEDDS enhances the levels of the antioxidant enzymes in the injured tissue (Figure 9). The levels of the SOD and CAT were augmented in the CA-SNEDDS formulation, while no difference in oxidative stress marker MPO or antioxidant GPx was observed after 3 weeks. Further studies with reduced duration of skin burn evaluation after 1 and 2 weeks and their comparison might indicate the relation of GPx and MPO levels during the wound healing process. In addition, CA-SNEDDS also demonstrated a reduction in the proinflammatory reduction marker, NAP3, in the wound zone. The wound healing potential of CA after 3 weeks of skin burn can be attributed to its antioxidant and anti-inflammatory effects. However, further studies are recommended to investigate the molecular mechanism of CA at the gene expression level of different vascular and proangiogenic/anti-inflammatory biomarkers involved in different phases of the skin wound healing process, in addition to concomitant systemic administration of CA along with the topical application.

## Figures and Tables

**Table 1:** Preliminary antimicrobial activity of CA-SNEDDS.

Microorganisms	IZD (Mean ± SD)			
	CA-SNEDDS	SNEDDS	Positive Control	Negative Control
**Gram-Positive Bacteria**				
*Staphylococcus aureus (S. aureus*) ATCC 29213	15.0 ± 0.2	6.0 ± 0.0	19.0 ± 0.3	6.0 ± 0.0
*Methicillin-resistantS. aureus* (MRSA) *	17.0 ± 0.3	6.0 ± 0.0	21.0 ± 0.2	6.0 ± 0.0
*Staphylococcus aureus* (*S. saptophyticus*) ATCC 43867	8.0 ± 0.9	6.0 ± 0.0	10.0 ± 0.3	6.0 ± 0.0
*Bacillus cereus* (*B. cereus*) ATCC 10876	8.0 ± 0.5	6.0 ± 0.0	10.0 ± 0.2	6.0 ± 0.0
**Gram-Negative Bacteria**				
*Escherichia coli* (*E. coli*) ATCC 25922	13.0 ± 0.5	6.0 ± 0.0	15.0 ± 0.1	6.0 ± 0.0
*Klebsiella pneumoniae* (*K. pneumoniae*) ATCC 27736	11.0 ± 0.5	6.0 ± 0.0	12.0 ± 0.3	6.0 ± 0.0
*Pseudomonas aeruginosa* (*P. aeruginosa*) ATCC 9027	8.0 ± 0.5	6.0 ± 0.0	10.0 ± 0.3	6.0 ± 0.0
*Salmonella typhimurium* (*S. typhimurium*) ATCC 13311	19.0 ± 0.9	6.0 ± 0.0	21.0 ± 0.3	6.0 ± 0.0
**Fungal Strains**				
*Candida albicans* (*C. albicans*) ATCC 10231	35.0 ± 0.5	6.0 ± 0.0	40.0 ± 0.1	6.0 ± 0.0
*Aspergillus niger* (*A. niger*) ATCC 6275	34.0 ± 0.5	6.0 ± 0.0	42.0 ± 0.4	6.0 ± 0.0

* Clinical isolate.

**Table 2:** MIC, MBC, MBIC, and MBEC of CA-SNEDDS.

Microorganisms	MIC	MBC	MBIC	MBEC
**Gram-Positive Bacteria**				
*S. aureus* ATCC 29213	6.25	12.5	12.5	25
MRSA	3.125	6.25	6.25	12.5
*S. saptophyticus* ATCC 43867	3.125	6.25	6.25	12.5
*B. cereus* ATCC 10876	6.25	12.5	12.5	25
**Gram-Negative Bacteria**				
*E. coli* ATCC 25922	3.125	6.25	6.25	12.5
*K. pneumoniae* ATCC 27736	3.125	6.25	6.25	12.5
*P. aeruginosa* ATCC 9027	3.125	6.25	6.25	12.5
*S. typhimurium* ATCC 13311	3.125	6.25	6.25	12.5
**Fungal Strains**				
*C. albicans* ATCC 10231	1.56	3.125	3.125	6.25
*A. niger* ATCC 6275	1.56	3.125	3.125	6.25

Note: All results are in µL/mL.

**Table 3:** Antioxidant activity of the tested compounds.

Compounds	TAC	DPPH-SA
CA	18.16 ± 0.84	6.60 ± 0.03
CA-SNEDDS	17.21 ± 0.72	6.57 ± 0.05

Note: All the methods were conducted in triplicate. TAA= Total antioxidant activity in mg Trolox equivalent. DPPH-SA, 2,2-diphenyl-1-picrylhydrazyl scavenging activity in mg Trolox.

## Data Availability

Data are available in the manuscript and Supplementary File.

## Supplementary Materials

The following supporting information can be downloaded at: https://www.mdpi.com/article/10.3390/molecules27165225/s1, Figure S1: The particle size distribution of self-nanoemulsifying drug delivery system (SNEDDS); Figure S2: The particle size distribution of cinnamaldehyde-loaded self-nanoemulsifying drug delivery system (CA-SNEDD); Figure S3: The zeta potential of self-nanoemulsifying drug delivery system (SNEDDS); Figure S4: The zeta potential of cinnamaldehyde-loaded self-nanoemulsifying drug delivery system (CA-SNEDDS); Table S1: One-way ANOVA for CA-SNEDDS; Table S2: Raw data of CAT assay; Table S3: Raw data of SOD assay; Table S4: Raw data of NAP3 assay; Table S5: Raw data of MPO assay.

## Sample Availability

The samples are available from the authors on request.
