# Supplementary material for: Cinnamaldehyde-Based Self-Nanoemulsion (CA-SNEDDS) Accelerates Wound Healing and Exerts Antimicrobial, Antioxidant, and Anti-Inflammatory Effects in Rats’ Skin Burn Model
Source: Molecules. 2022 Aug 16;27(16):5225. doi: 10.3390/molecules27165225 (PMC9413107; doi:10.3390/molecules27165225)
Supplement: Supplementary file 1 [file molecules-27-05225-s001.zip › molecules-1814130-supplementary.pdf]

Supplementary Material

Results

			Size (d.n...	% Intensity:	St Dev (d.n...
Z-Average (d.nm):	31.59	Peak 1:	20.86	53.8	9.059
PdI:	0.651	Peak 2:	200.9	43.8	103.8
Intercept:	0.740	Peak 3:	4435	2.4	910.0
Result quality	Good				

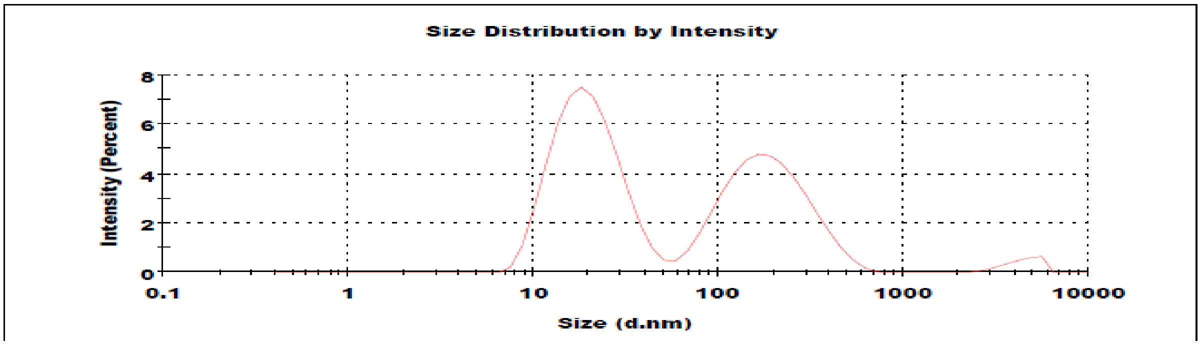

Figure S1. The particle size distribution of self-nano emulsifying drug delivery system (SNEDDS).

Results

			Size (d.n...	% Intensity:	St Dev (d.n...
Z-Average (d.nm):	38.63	Peak 1:	48.27	88.8	39.78
PdI:	0.431	Peak 2:	360.4	6.8	117.6
Intercept:	0.851	Peak 3:	5075	4.4	568.7
Result quality	Good				

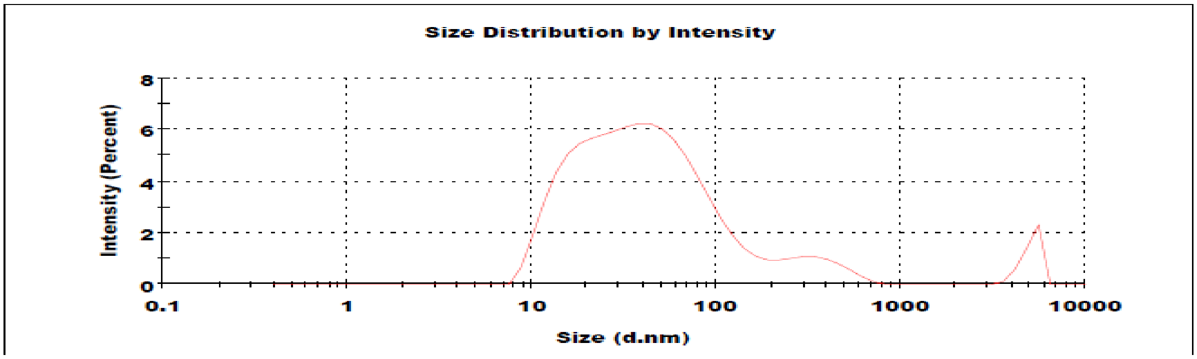

Figure S2. The particle size distribution of cinnamaldehyde loaded self-nano emulsifying drug delivery system (CA-SNEDD.

Results

			Mean (mV)	Area (%)	St Dev (mV)
Zeta Potential (mV):	-9.70	Peak 1:	-12.7	62.7	4.97
Zeta Deviation (mV):	6.62	Peak 2:	-3.46	37.3	2.93
Conductivity (mS/cm):	0.0603	Peak 3:	0.00	0.0	0.00
Result quality	Good				

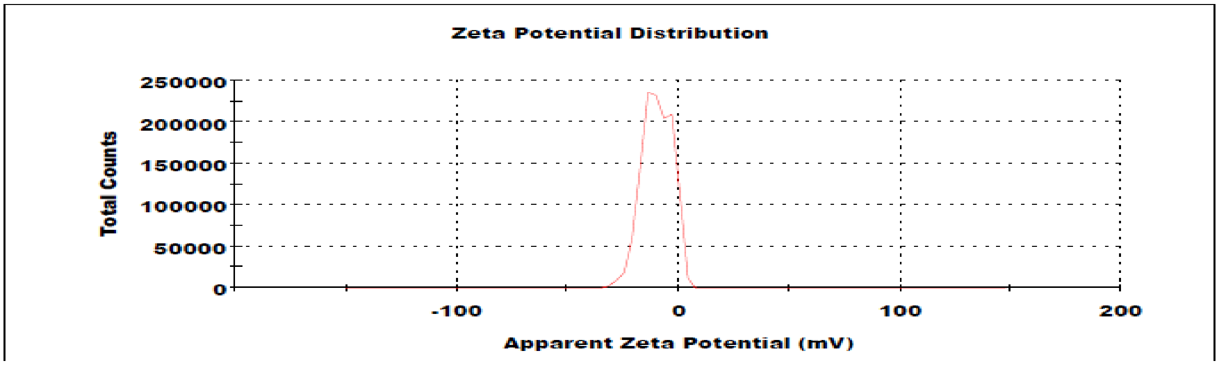

Figure S3. The zeta potential of self-nano emulsifying drug delivery system (SNEDDS).

Results

			Mean (mV)	Area (%)	St Dev (mV)
Zeta Potential (mV):	-9.90	Peak 1:	-9.90	100.0	4.10
Zeta Deviation (mV):	4.10	Peak 2:	0.00	0.0	0.00
Conductivity (mS/cm):	0.0731	Peak 3:	0.00	0.0	0.00
Result quality	Good				

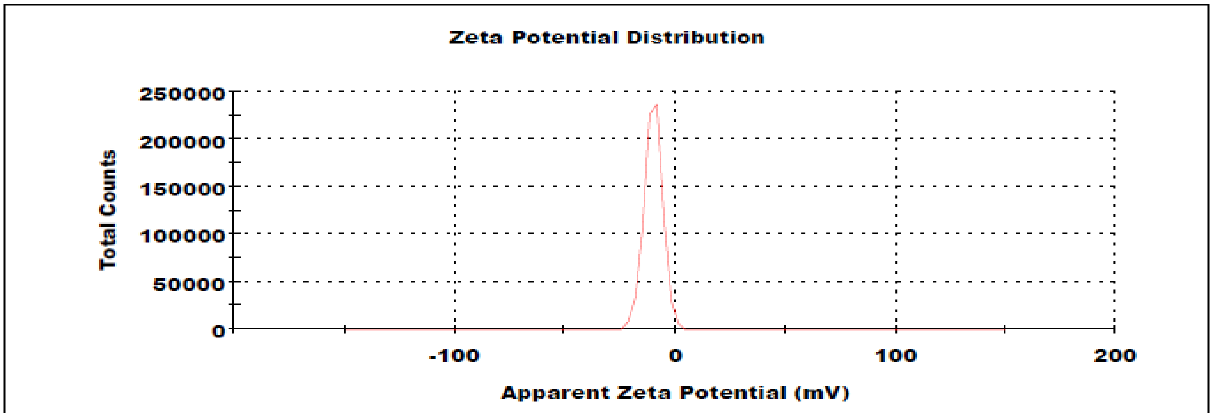

Figure S4. The zeta potential of cinnamaldehyde loaded self-nano emulsifying drug delivery system (CA-SNEDDS).

Table S1: One-way ANOVA for CA-SNEDDS.

ANOVA					
CA-SNEDDS					
	Sum of Squares	df	Mean Square	F	Sig.
Between Groups	2746.800	9	305.200	975.080	.000
Within Groups	6.260	20	.313		
Total	2753.060	29			

Table S2: Raw data of CAT assay

CAT

	Negative control	SNEDDS control	Silver Sulfadiazine	Cinnamaldehyde SNEDDS
	0.298	0.367	0.373	0.337
	0.297	0.347	0.324	0.372
	0.312	0.304	0.398	0.359
	0.317	0.309	0.338	0.321
	0.321	0.361	0.35	0.396
**Average**	**0.309**	**0.338**	**0.357**	**0.357**
**median**	**0.312**	**0.347**	**0.350**	**0.359**
**S.D**	**0.011**	**0.029**	**0.029**	**0.029**
**n**	**5.000**	**5.000**	**5.000**	**5.000**
**S.E**	**0.005**	**0.013**	**0.013**	**0.013**

Table S3: Raw data of SOD assay

SOD

Negative control	SNEDDS control	Silver Sulfadiazine	Cinnamaldehyde SNEDDS
0.207591	0.15004	0.217694	0.209622
0.184462	0.171549	0.220189	0.201746
0.139786	0.116436	0.239262	0.182252
0.11392	0.118086	0.196276	0.20278
0.086867	0.09334	0.208863	0.21944

**0.147**	**0.130**	**0.216**	**0.203**
**0.140**	**0.118**	**0.218**	**0.203**
**0.050**	**0.031**	**0.016**	**0.014**
**5.000**	**5.000**	**5.000**	**5.000**
**0.022**	**0.014**	**0.007**	**0.006**

Table S4: Raw data of NAP3 assay

NAP3

Negative control	SNEDDS control	Silver Sulfadiazine	Cinnamaldehyde SNEDDS
35.59333	37.0441	29.02667	30.39333
33.72667	36.02667	28.26	32.62667
67.29333	42.22667	31.39333	33.56
50.99333	43.02667	30.96	36.29333
41.19333	39.22667	29.89333	30.76
**45.760**	**39.510**	**29.907**	**32.727**
**41.193**	**39.227**	**29.893**	**32.627**
**13.785**	**3.084**	**1.304**	**2.384**
**5.000**	**5.000**	**5.000**	**5.000**
**6.165**	**1.379**	**0.583**	**1.066**

Table S5: Raw data of MPO assay

MPO

Negative control	SNEDDS control	Silver Sulfadiazine	Cinnamaldehyde SNEDDS
0.253495	0.253495	0.252889	0.251332
0.25237	0.252457	0.251419	0.251592
0.257734	0.25436	0.253235	0.25237
0.252803	0.25263	0.251419	0.252543
0.25237	0.251246	0.252197	0.252457
**0.254**	**0.253**	**0.252**	**0.252**
**0.253**	**0.253**	**0.252**	**0.252**
**0.002**	**0.001**	**0.001**	**0.001**
**5.000**	**5.000**	**5.000**	**5.000**
**0.001**	**0.001**	**0.000**	**0.000**
